# Parkinsonism-Hyperpyrexia Syndrome in a patient undergoing upper gastrointestinal surgery: A case report

**DOI:** 10.5339/qmj.2023.34

**Published:** 2024-01-10

**Authors:** Nissar Shaikh, Adnan Saadeddin

**Affiliations:** 1 Department of Anesthesia and Perioperative Care, Hamad Medical Corporation, Doha, Qatar Email: saadeddinmd@gmail.com ORCID iD: 0000-0002-9718-949X

**Keywords:** Parkinson’s disease, antiparkinsonian medications, perioperative management, neuroleptic malignant syndrome, parkinsonism-hyperpyrexia syndrome.

## Abstract

Background: Parkinsonism-hyperpyrexia syndrome (PHS) is a potentially life-threatening condition that occurs due to the abrupt withdrawal or significant dose reduction of antiparkinsonian medications. It presents similarly to neuroleptic malignant syndrome (NMS) and is characterized by severe rigidity, fever, autonomic instability, and altered mental status.

Case: A 62-year-old male with a 10-year history of Parkinson’s disease (PD) underwent laparoscopic mesh repair for a left-sided diaphragmatic and large hiatus hernia. His antiparkinsonian medications included levodopa/carbidopa, amantadine, pramipexole, and benzhexol. Medications were withheld as part of the nil per os (NPO) status. Postoperatively, he developed withdrawal features, including tremors, difficulty speaking, tachycardia, hypertension, fever, and sweating. PHS, resulting from the withdrawal of antiparkinsonian medications, was diagnosed. The patient was transferred to the intensive care unit (ICU), intubated, and his antiparkinsonian medications were reintroduced. The patient’s condition improved gradually, and he was discharged home on the 15th postoperative day.

Discussion: The abrupt discontinuation of antiparkinsonian medications precipitated PHS in our patient. Recognizing the clinical picture of PHS and differentiating it from other possible conditions, such as neuroleptic malignant syndrome and malignant hyperthermia, is pivotal. Management involves resuming medications and providing supportive care. Early recognition and prompt reintroduction of the antiparkinsonian medications are essential for the patient’s recovery.

Conclusion: PHS is a rare but potentially life-threatening condition that occurs due to the withdrawal of antiparkinsonian medications, leading to an acute hypodopaminergic state. Our case emphasizes the importance of careful perioperative management of antiparkinsonian medications and early recognition and management of withdrawal symptoms in patients with Parkinson’s disease undergoing surgery.

## Introduction

Parkinson’s disease (PD) is a progressive neurodegenerative disorder resulting from basal ganglia dysfunction. It is considered a movement disorder characterized by bradykinesia, resting tremors, rigidity, and postural instability. Non-motor symptoms include autonomic dysfunction, cognitive impairment, and psychiatric symptoms.^1^ Levodopa is the most commonly used drug for the treatment of Parkinson’s disease. Other medications used include amantadine, non-ergot dopamine agonists (e.g., pramipexole, ropinirole), monoamine oxidase (MAO) inhibitors (e.g., selegiline, rasagiline), catechol-O-methyltransferase inhibitors (e.g., entacapone), and anticholinergics (e.g., benztropine).^2^

Perioperative management of PD might be challenging due to the possibility of withdrawal features from antiparkinsonian medications. The constellation of features occurring due to the withdrawal of the antiparkinsonian medications is referred to in the literature as parkinsonism-hyperpyrexia syndrome (PHS) or neuroleptic malignant-like syndrome.^3,4^ Our case describes a patient with PD who developed PHS following upper gastrointestinal surgery due to the sudden withdrawal of his medications. Informed written consent and institutional approval (MRC-04-23-420) to publish this case report have been obtained.

## Case Summary

A 62-year-old man with a medical history of Parkinson’s disease for 10 years, hypertension for 10 years, and hypothyroidism for 6 years presented with a 6-month history of shortness of breath, cough with sputum, and chest cramps after walking. Preoperative investigations, including computed tomography (CT) thorax with contrast and esophagogastroduodenoscopy (OGD), revealed a left-sided diaphragmatic hernia and a large hiatus hernia with a significant portion of the stomach herniated and convoluted inside. The patient was scheduled to undergo laparoscopic mesh repair at a tertiary government-funded hospital.

His antiparkinsonian medications included levodopa/carbidopa 100 mg/25 mg four times daily, amantadine 100 mg twice daily, pramipexole 1.5 mg daily, and benzhexol 2 mg four times daily. He was also treated with losartan 100 mg daily and amlodipine 10 mg daily for hypertension, and levothyroxine 100 mcg daily for hypothyroidism. Benzhexol and pramipexole were started 10 years ago at the time of diagnosis. Amantadine was started one year after the diagnosis, and levodopa/carbidopa was initiated two years after the diagnosis.

On the morning of the surgery, the antiparkinsonian medications were withheld, and the patient underwent an 8-hour uneventful laparoscopic mesh repair of the paraesophageal hernia. The intraoperative surgical and anesthetic courses were uneventful. Intraoperatively, induction was achieved using propofol 150 mg, fentanyl 100 mcg, ketamine 50 mg, lidocaine 100 mg, and rocuronium 100 mg. Maintenance of anesthesia was achieved with the inhalational anesthetic desflurane and remifentanil infusion. Other medications received intraoperatively included dexamethasone 8 mg, calcium gluconate 2 grams, phenylephrine 200 mcg, cefuroxime (a total of 3000 mg), metronidazole (a total of 1000 mg), paracetamol 1000 mg, ondansetron 4 mg, morphine 10 mg, and ketorolac 30 mg.

On the day following the day of surgery (the first postoperative day), levodopa/carbidopa was resumed approximately 38 hours after the last preoperative dose. However, benzhexol, pramipexole, and amantadine were not resumed as the patient was still in nil per os (NPO) status and oral intake usually commenced gradually after upper gastrointestinal surgeries.

On the second postoperative day, the patient reported feeling weak and developed excessive left arm tremor, an inability to clear oral secretions with a weak cough, and difficulty speaking. He also experienced tachycardia, hypertension, and sweating, as well as one episode of fever (38.1°C). His heart rate reached 140 beats per minute, respiratory rate 34 breaths per minute, and blood pressure 206/111 mmHg. His oxygen saturation (SpO2) was only 92% on oxygen 10 L/min via facemask.

A neurological examination revealed a drowsy but arousable patient with excessive left arm tremors. He had generalized cogwheel rigidity. No lateralization signs were evident, and muscle power was 5/5 in all limbs. It was suspected that the patient was suffering from dopaminergic withdrawal due to withholding his antiparkinsonian medications. The patient was intubated because of the decreased level of consciousness, weak cough, inability to clear secretions, and desaturation. Hence, he was transferred to the intensive care unit (ICU), with the remainder of his antiparkinsonian medications reintroduced approximately 68 hours after the last dose. The patient received propofol and remifentanil infusions for sedation and required a low-dose noradrenaline infusion for hemodynamic support. The ventilator mode used was continuous mandatory ventilation (CMV) with pressure control (14 cmH2O) and FiO2 (50%). At this time, laboratory tests showed elevated levels of Creatine Kinase (CK) at 560 U/L, Alanine Aminotransferase (ALT) at 350 U/L, Aspartate Aminotransferase (AST) at 260 U/L, Urea levels at 9.9 mmol/L (27.7 mg/dL), and a thyroid-stimulating hormone (TSH) level of 1.87 mlU/L. C-reactive protein (CRP) increased to 110 mg/dL and trended down later. Blood, sputum, and urine cultures were all negative. A chest x-ray revealed bilateral pleural effusion with basal consolidations.

**Figure 1.**
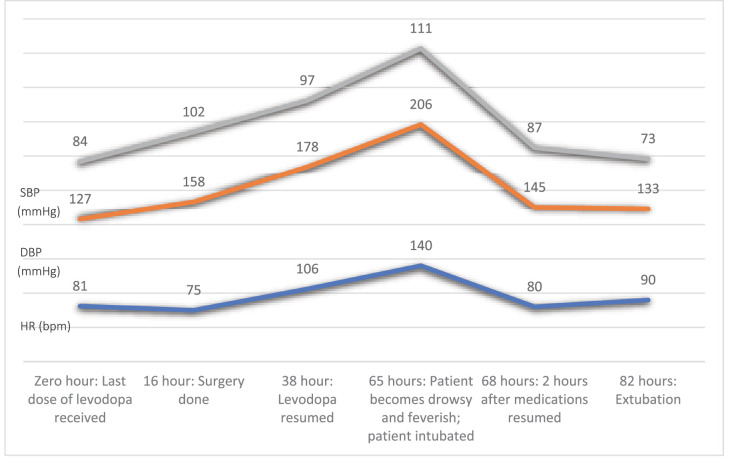
Hemodynamic changes throughout the perioperative course, where the x-axis represents the perioperative events, and the y-axis represents the hemodynamic values. Zero hour is the time the last dose of levodopa was received preoperatively. Subsequent time points are the number of hours elapsed since the last dose of levodopa. (SBP: Systolic blood pressure, DBP: Diastolic blood pressure, HR: heart rate, bpm: beats per minute).

Despite resuming his antiparkinsonian medications, the patient remained intubated and ventilated and required vasopressor support and continuous monitoring. The patient was eventually extubated on the third postoperative day, with continued support through incentive spirometry, chest physiotherapy, and high-flow nasal cannula (HFNC) oxygen. Figure 1 illustrates the changes in the patient’s systolic and diastolic blood pressure and heart rate from the last dose of levodopa received to 82 hours post-operation. Over the subsequent days, the patient’s condition gradually improved. His vital signs stabilized, and his CK, ALT, AST, and Urea started trending toward normalization. He was shifted to the general ward on the 9th postoperative day and discharged home on the 15th postoperative day.

## Discussion

We present a Parkinson’s disease patient who underwent upper gastrointestinal surgery, was kept NPO for 2 days, and developed severe withdrawal features approximately 65 hours after stopping the antiparkinsonian medications perioperatively. The patient reported feeling weak and developed excessive tremors, inability to swallow, difficulty speaking, sweating, and fever associated with tachycardia and hypertension. This presentation is consistent with the diagnosis of PHS. The patient required intubation and ICU care but gradually improved and was discharged home 15 days postoperatively.

PHS is a rare but potentially life-threatening complication observed in patients with PD, usually due to the abrupt cessation of antiparkinsonian medications.^5^ The presentation of PHS is generally similar to neuroleptic malignant syndrome (NMS), including high fever, muscle rigidity, decreased level of consciousness, dysautonomia, leukocytosis, and high creatine kinase (CK).^3^

The main differential diagnoses are neuroleptic malignant syndrome (due to dopamine antagonists) and serotonin syndrome. Malignant hyperthermia is a possible differential diagnosis; however, this condition usually occurs intraoperatively and less frequently in the immediate postoperative period.^6^ The patient described in this case developed the clinical features on the second postoperative day, making the malignant hyperthermia diagnosis extremely unlikely. Other differential diagnoses include malignant catatonia and dyskinesia-hyperpyrexia syndrome. Understanding the clinical context in which these features occur is essential for diagnosing properly.^7^ In this case, the patient developed pleural effusion, which might be a possible cause of the desaturation. However, it doesn’t explain the constellation of features described, particularly the neurological features. There are no standardized diagnostic criteria specific to PHS, but an international consensus group published diagnostic criteria for NMS in 2011. The criteria included 1) exposure to dopamine antagonist or withdrawal of dopamine agonist within 72 hours, 2) hyperthermia, 3) rigidity, 4) altered mental status, 5) elevated CK, 6) sympathetic lability defined by blood pressure elevation, diaphoresis, or urinary incontinence, 7) hypermetabolism defined by increased heart rate and respiratory rate, and 8) exclusion of infectious, toxic, metabolic, or neurologic causes.^8^

The central pathophysiological mechanism for PHS is a central hypodopaminergic state, resulting in a sudden reduction in dopaminergic input to the basal ganglia and other nervous system structures, exacerbating the preexisting dopamine deficit.^3^ Complications of PHS can be severe and life-threatening, including acute renal failure, rhabdomyolysis, aspiration pneumonia, deep vein thrombosis, and disseminated intravascular coagulation.^5^

In our case, PHS was precipitated by the abrupt discontinuation of the antiparkinsonian medications. Patients undergoing gastrointestinal surgery may require staying NPO for a prolonged period, like our patient, which may make it challenging to manage the antiparkinsonian medications perioperatively.

Antiparkinsonian medications should be continued as perioperatively as possible, with the morning dose taken before the surgery and the regimen resumed as soon as feasible postoperatively.^9^ For long surgeries or for patients who are expected to stay NPO for a prolonged time, weaning off antiparkinsonian medications preoperatively might reduce the risk of withdrawal features.^9^ However, there are no protocols to inform when and how to reduce the dose.^10^ Alternatively, parenteral antiparkinsonian regimens are available and might be used in these conditions. These include intravenous (IV) levodopa,^10,11^ IV amantadine,^12^ transdermal rotigotine,^13^ and subcutaneous apomorphine and rectal domperidone.^14^

There are similar cases reported in the literature where patients developed similar features after withdrawing or reducing the dosages of their antiparkinsonian medications.^15,16,17^ Similar presentations were also reported following the withdrawal or malfunction of deep brain stimulation devices.^3,7^ Reintroducing the medications and providing supportive care led to improvements in the clinical condition within 15 hours to 2 weeks in most reported cases, consistent with our patient’s recovery. However, the mortality rate of PHS has been reported to be 4% when treated and may reach up to 20% when untreated.^9^ Compared to the cases described in the literature, our patient had similar precipitating factors, clinical presentation, and management strategy. Our patient had only one episode of fever, in contrast to multiple reported cases where patients had more severe fever.^3,7^

This case emphasizes the importance of careful management of the antiparkinsonian medications perioperatively, as it demonstrates the potential for severe withdrawal symptoms and complications. Parkinson’s disease patients are highly sensitive to changes in medication timing, and it is crucial to maintain consistency with their home regimen through precise medication reconciliation that focuses on administration time. Before surgery, patients should be asked about the usual effects of missed or delayed dosing, including how quickly they notice breakthrough neuromuscular symptoms and their flexibility with dosing windows.^9^ Clinicians should be aware of the risks associated with withholding antiparkinsonian medications and take individual patient factors into account when making perioperative medication management decisions. Clinicians should also consider the possibility of changes in the pharmacokinetics of levodopa in patients undergoing gastrointestinal surgeries, which may affect the efficacy of levodopa even beyond the perioperative period.^18,19^ Understanding the pharmacokinetics of the medications helps make decisions regarding the management of the antiparkinsonian medications perioperatively. levodopa/carbidopa has a relatively short half-life of approximately 90 minutes compared to the other medications used in this patient.^20^ The plasma half-life of amantadine has averaged 16 ± 6 hours,^21^ and that of pramipexole is approximately 8 to 12 hours. A much longer half-life is observed with benzhexol, with a half-life lasting as long as 33 hours.^22^

The case also highlights the challenges in the diagnosis and management of parkinsonism-hyperpyrexia syndrome. Early recognition and prompt reintroduction of the antiparkinsonian medications are essential to the patient’s recovery. Effective communication among the healthcare team, including anesthesiologists, surgeons, and neurologists, is crucial in the management of patients with Parkinson’s disease undergoing surgery. Patient education on the potential complications and the importance of adhering to their treatment regimen can help minimize the risk of PHS.

Healthcare providers should focus on developing guidelines for the perioperative management of patients with Parkinson’s disease, particularly those taking antiparkinsonian medications or receiving deep brain stimulation therapy. Further research is needed to explore the risk factors of PHS in the perioperative setting to help anesthesiologists and surgeons identify patients at risk and guide their management.

## Conclusion

This case report describes a patient with Parkinson’s disease who underwent upper gastrointestinal surgery and subsequently developed PHS due to the abrupt discontinuation of his antiparkinsonian medications. He required admission to the ICU for mechanical ventilation and hemodynamic support. The patient eventually recovered well and was discharged home after a prolonged inpatient course. This case also highlights the significance of careful perioperative management of antiparkinsonian medications in patients with Parkinson’s disease undergoing upper gastrointestinal surgery. Abrupt discontinuation of these medications can lead to severe withdrawal symptoms, as observed in our patient who developed PHS. Early recognition and prompt reintroduction of antiparkinsonian medications are crucial for patient recovery. This case highlights the need for developing comprehensive guidelines for perioperative management in patients with Parkinson’s disease.

## Abbreviations

ALT (alanine transaminase)

AST (aspartate transaminase)

CK (Creatine Kinase)

CMV (Continuous Mandatory Ventilation)

CRP (C-Reactive Protein)

CT (computed tomography)

FiO_2_ (Fraction of Inspired Oxygen)

HFNC (high-flow nasal cannula)

ICU (intensive care unit)

IV (Intravenous)

MAO (Monoamine Oxidase)

NMS (neuroleptic malignant syndrome)

NPO (Nil Per Os)

OGD (esophagogastroduodenoscopy)

PD (Parkinson’s Disease)

PHS (Parkinsonism-Hyperpyrexia Syndrome)

SpO_2_ (Oxygen Saturation)

TSH (Thyroid Stimulating Hormone)

## Conflicts of Interest

The authors declared that there were no competing interests.

## Funding Sources

This work still needs to receive funding.

## References

[1] Bandopadhyay R, Mishra N, Rana R, Kaur G, Ghoneim MM, Alshehri S (2022). Molecular Mechanisms and Therapeutic Strategies for Levodopa-Induced Dyskinesia in Parkinson’s Disease: A Perspective Through Preclinical and Clinical Evidence. Front Pharmacol.

[2] Armstrong MJ, Okun MS (2020). Diagnosis and Treatment of Parkinson Disease: A Review. JAMA.

[3] Grover S, Sathpathy A, Reddy SC, Mehta S, Sharma N (2018;). Parkinsonism-hyperpyrexia syndrome: A case report and review of literature. Indian J Psychiatry.

[4] Toru M, Matsuda O, Makiguchi K, Sugano K (1981). Neuroleptic malignant syndrome-like state following a withdrawal of antiparkinsonian drugs. J Nerv Ment Dis.

[5] Newman EJ, Grosset DG, Kennedy PGE (2009;). The parkinsonism-hyperpyrexia syndrome. Neurocrit Care.

[6] Litman RS, Flood CD, Kaplan RF, Kim YL, Tobin JR (2008). Postoperative Malignant Hyperthermia: An Analysis of Cases from the North American Malignant Hyperthermia Registry. Anesthesiology.

[7] Azar J, Jaber Y, Ayyad M, Abu Alia W, Owda F, Sharabati H (2022). Parkinsonism-Hyperpyrexia Syndrome: A Case Series and Literature Review. Cureus.

[8] Gurrera RJ, Caroff SN, Cohen A, Carroll BT, DeRoos F, Francis A (2011). An international consensus study of neuroleptic malignant syndrome diagnostic criteria using the Delphi method. J Clin Psychiatry.

[9] Oprea AD, Keshock MC, O’Glasser AY, Cummings KC, Edwards AF, Hunderfund AL (2022). Preoperative Management of Medications for Neurologic Diseases: Society for Perioperative Assessment and Quality Improvement Consensus Statement. Mayo Clinic Proceedings.

[10] Fujii T, Nakabayashi T, Hashimoto S, Kuwano H (2009;). Successful perioperative management of patients with Parkinson’s disease following gastrointestinal surgery: report of three cases. Surg Today.

[11] Mizuno J, Kato S, Watada M, Morita S (2009). [Perioperative management of a patient with Parkinson’s disease with intravenous infusion of levodopa]. Masui.

[12] Kim YE, Kim HJ, Yun JY, Jeon BS (2011). Intravenous amantadine is safe and effective for the perioperative management of patients with Parkinson’s disease. J Neurol.

[13] Wüllner U, Kassubek J, Odin P, Schwarz M, Naumann M, Häck HJ (2010;). Transdermal rotigotine for the perioperative management of Parkinson’s disease.

[14] Gálvez-Jiménez N, Lang AE (1996). Perioperative problems in Parkinson’s disease and their management: apomorphine with rectal domperidone. Can J Neurol Sci.

[15] Akcali A, Savas L (2008). Malignant syndrome of two Parkinson patients due to withdrawal of drugs. Ann Acad Med Singap.

[16] Cheung YF, Hui CHT, Chan JHM (2011). Parkinsonism-hyperpyrexia syndrome due to abrupt withdrawal of amantadine. Hong Kong Med J.

[17] Arora A, Fletcher P (2013). arkinsonism hyperpyrexia syndrome caused by abrupt withdrawal of ropinirole. Br J Hosp Med (Lond).

[18] Nagayama H, Kajimoto Y, Kumagai T, Nishiyama Y, Mishina M, Kimura K (2015;). Pharmacokinetics of Levodopa before and after Gastrointestinal Resection in Parkinson’s Disease. CRN.

[19] Miyaue N, Yabe H, Nagai M, Nomoto M (2020). Gastrointestinal surgical procedures affect levodopa pharmacokinetics in Parkinson’s disease. Parkinsonism Relat Disord.

[20] Nyholm D, Odin P, Johansson A, Chatamra K, Locke C, Dutta S (2012). Pharmacokinetics of Levodopa, Carbidopa, and 3-O-Methyldopa Following 16-hour Jejunal Infusion of Levodopa-Carbidopa Intestinal Gel in Advanced Parkinson’s Disease Patients. AAPS J.

[21] Aoki FY, Sitar DS (1988). Clinical pharmacokinetics of amantadine hydrochloride. Clin Pharmacokinet.

[22] Deleu D, Northway MG, Hanssens Y (2002;). Clinical pharmacokinetic and pharmacodynamic properties of drugs used in the treatment of Parkinson’s disease. Clin Pharmacokinet.

